# Telomerase Inhibition Decreases Alpha-Fetoprotein Expression and Secretion by Hepatocellular Carcinoma Cell Lines: *In Vitro* and *In Vivo* Study

**DOI:** 10.1371/journal.pone.0119512

**Published:** 2015-03-30

**Authors:** Roula Tahtouh, Anne-Sophie Azzi, Nada Alaaeddine, Soulaima Chamat, Hasnaa Bouharoun-Tayoun, Layal Wardi, Issam Raad, Riad Sarkis, Najibe Abou Antoun, George Hilal

**Affiliations:** 1 Cancer and Metabolism Laboratory, Faculty of Medicine, Saint-Joseph University, Beirut, Lebanon; 2 Regenerative Medicine Laboratory, Faculty of Medicine, Saint-Joseph University, Beirut, Lebanon; 3 Faculty of Health Sciences, Lebanese University, Fanar, Lebanon; 4 Department of Infectious Diseases, the University of Texas M. D. Anderson Cancer Center, Houston, Texas, United States of America; 5 Faculty of Medicine, Saint-Joseph University and Hotel-Dieu de France, Surgery Department, Beirut, Lebanon; University of Medicine, Greifswald, Germany, GERMANY

## Abstract

Alpha-fetoprotein (AFP) is a diagnostic marker for hepatocellular carcinoma (HCC). A direct relationship between poor prognosis and the concentration of serum AFP has been observed. Telomerase, an enzyme that stabilizes the telomere length, is expressed by 90% of HCC. The aim of this study was to investigate the effect of telomerase inhibition on AFP secretion and the involvement of the PI3K/Akt/mTOR signaling pathway. Proliferation and viability tests were performed using tetrazolium salt. Apoptosis was determined through the Annexin V assay using flow cytometry. The concentrations of AFP were measured using ELISA kits. The AFP mRNA expression was evaluated using RT-PCR, and cell migration was evaluated using a Boyden chamber assay. The in vivo effect of costunolide on AFP production was tested in NSG mice. Telomerase inhibition by costunolide and BIBR 1532 at 5 and 10 μM decreased AFP mRNA expression and protein secretion by HepG2/C3A cells. The same pattern was obtained with cells treated with hTERT siRNA. This treatment exhibited no apoptotic effect. The AFP mRNA expression and protein secretion by PLC/PRF/5 was decreased after treatment with BIBR1532 at 10 μM. In contrast, no effect was obtained for PLC/PRF/5 cells treated with costunolide at 5 or 10 μM. Inhibition of the PI3K/Akt/mTOR signaling pathway decreased the AFP concentration. In contrast, the MAPK/ERK pathway appeared to not be involved in HepG2/C3A cells, whereas ERK inhibition decreased the AFP concentration in PLC/PRF/5 cells. Modulation of the AFP concentration was also obtained after the inhibition or activation of PKC. Costunolide (30 mg/kg) significantly decreased the AFP serum concentration of NSG mice bearing HepG2/C3A cells. Both the inhibition of telomerase and the inhibition of the PI3K/Akt/mTOR signaling pathway decreased the AFP production of HepG2/C3A and PLC/PRF/5 cells, suggesting a relationship between telomerase and AFP expression through the PI3K/Akt/mTOR pathway

## Introduction

Hepatocellular carcinoma (HCC) is the fifth most common human cancer and is the third highest cause of cancer mortality worldwide [1]. The etiology of HCC has been reported to be linked to several types of diseases, such as chronic hepatitis C [2], alcoholic hepatitis [3], non-alcoholic steatohepatitis (NASH) [4], diabetes mellitus and metabolic syndrome [5]. The diagnostic strategies for identifying early HCC remain the subject of many studies and are defined by the tumor size and the number of lesions [6]. The most common methods used to diagnose HCC are radiographic imaging, liver biopsy and measurement of the serum tumor marker alpha-fetoprotein (AFP) [6].

AFP is a 70-kD glycoprotein consisting of 591 amino acids [7] encoded by a gene on chromosome 4q11-q13 [8]. Normally synthesized by the fetal liver, yolk sac and the tissue of gastrointestinal system [9], AFP is highly elevated at the age of 10 to 13 weeks, and its levels decrease during gestation [10]. Altered levels of maternal and fetal AFP have been associated with birth defects, including hypothyroidism, autoimmune diseases and heart defects [8]. In addition, AFP is used as a marker for the evaluation and diagnosis of Down’s syndrome and neural tube defect [11,12]. Another common use of AFP is the confirmation and monitoring of certain pathological conditions, including hepatoblastoma, hepatocellular carcinoma, germ cell cancer and gastric cancers [13,14]. Moreover, AFP can also be expressed in benign conditions, such as active hepatitis and cirrhosis [15,16]. The concentration of this glycoprotein is actually measured by two-site immunometric assays using monoclonal and/or polyclonal antibodies [17]. In addition to its extended use as a marker for the clinical diagnosis of HCC, the physiological and pathological roles of AFP have currently prompted interest due to its close association with carcinogenesis [18]. As a member of the albumin family, AFP acts as a binding protein and transports steroid hormones, bilirubin, fatty acids and retinoids [19].

The AFP gene is regulated by several transcription factors, such as fetoprotein transcription factors [20], promoter coupling factor [21], HNF1 (hepatocyte nuclear factor) [22–24], NKx2.8 [25], C/EBP (CCAAT/enhancer-binding protein) [23], RXR and RAR receptors [26], and NF1 (nuclear factor 1) [23]. In fact, the AFP gene is differentially modulated by retinoic acid in tumor cell lines. However, it is downregulated by retinoic acid in the human hepatoma cell line HepG2 [27] and activated in other cell lines, such as the rat hepatoma cell line MCA-RH8994 and the teratocarcinoma stem cell line F9 [26]. The transcription of AFP is also suppressed by P53, which binds to the AFP repressor domain, through the inhibition of the HNF-3 activator [28]. In mice, Raf and Rif are involved in the regulation of AFP gene expression after birth [29,30]. In addition, AFP regulates cell proliferation and is involved in cell differentiation and growth regulation [31]. AFP is known to be involved in growth and apoptosis signal pathways [31]. The signaling pathway used by AFP remains unknown, even though a few studies have demonstrated the presence of a specific receptor for this protein on the cell surface that has the ability to be internalized by endocytosis [32]. Moreover, cytoplasmic AFP acts as a regulator for promoting the PI3K/Akt pathway by interfering with the PTEN protein in human HCC cells [13,14]. The PI3K/Akt signaling pathway regulates survival mechanisms, cycle progression and cell growth. In fact, phosphorylated Akt can regulate apoptosis and cell proliferation by targeting different factors, such as Bad, p27 and mTOR [33]. Once mTOR is phosphorylated, it catalyzes the phosphorylation of the transcription factor STAT3 [34]. The activation of STAT3 is related to several cancer types, such as prostate and breast cancer [35]. AFP is produced by the majority of hepatocellular carcinomas and is directly related to the severity of the disease [36]. Despite the expression of this marker in other malignancies, such as breast cancer, gastric cancer and germ cell cancer, it remains the only serum marker for the diagnosis of HCC because of the high expression of this protein in pathological liver tissue [16]. A recent study showed that the concentration of AFP in the serum is a marker for assessing the response to therapy and a marker of disease progression and survival in patients with hepatocellular carcinoma [37]. In addition to the secretion of AFP, 90% of hepatocellular carcinoma cells express telomerase.

Telomerase is an enzyme expressed by more than 85% of cancers and responsible for the immortalization of many cancer cells [38]. It stabilizes the telomere length, the ends of linear chromosomes and synthesizes *de novo* telomeric repeats that have been lost due to incomplete DNA replication, nucleolytic degradation and oxidative stress [39,40]. The maintenance of telomeres and telomerase plays an essential role in senescence, aging and cancer [41]. The two major components of human telomerase are reverse transcriptase (hTERT), catalytic subunit, and RNA subunit (hTR) [42]. The hTR consists of 451 nucleotides and is transcripted by RNA polymerase II. It acts as a template for telomeric DNA. The hTR gene was localized on chromosome 3q26.3 in 1998 [43]. In fact, the RNA subunit contains an 11-nucleotide single-stranded region known as the template region, which makes it accessible for hybridization with complementary nucleic acids, leading to hTR inhibition [44]. hTERT catalyzes the addition of telomeric repeats to the 3' ends of chromosomes. Human telomerase also contains several accessory proteins that bind to either hTERT or hTR, thereby forming a ribonucleoprotein complex with a large molecular weight of 1000 kDa [45]. Telomerase is also active in embryonic tissues, proliferating cells, activated lymphocytes and germ stem cells, but the enzyme activity in these cell types is much lower than that measured in cancer cells [46,47]. Telomerase activity is regulated by the hTERT subunit [48]. In fact, hTERT mRNA expression is suppressed by many factors, such as retinoblastoma protein (Rb), cyclin-dependent kinase inhibitor p21 and P53, in different cell lines, including glioma, breast cancer and squamous cell carcinoma [49,50]. Moreover, hTERT transcription and telomerase activity are upregulated by c-myc [51–53]. Furthermore, interleukin-6 (IL-6) and insulin-like growth factor-1 (IGF-1) induce telomerase activity in myeloma cell lines [54]. C/EBP also appears to be involved in the regulation of TERT promoter activity in tumor tissues [55]. Recently, the phosphorylation of hTERT protein has been reported to be involved in telomerase activity regulation. Additionally, protein phosphatase 2A (PP2A) has been reported to inhibit telomerase activity [56].

Several studies have shown a correlation between increased telomerase activity and the high level of AFP observed in hepatocellular carcinoma. The present study provides the first demonstration of an interrelationship between telomerase activity and AFP secretion. Furthermore, telomerase inhibition decreases AFP expression and secretion likely through the PI3K/Akt/mTOR/STAT3 pathway in the HCC cell lines HepG2/C3A and PLC/PRF/5, which express telomerase and AFP.

## Materials and Methods

### Cell culture

HepG2/C3A and PLC/PRF/5 HCC cells were purchased from ATCC (American Type Cell Culture, USA) and maintained as recommended. HepG2/C3A cells were cultured in DMEM (1g/L glucose) containing 10% fetal bovine serum, 1% penicillin/streptomycin (Sigma Chemical Co., St. Louis, MO, USA) and 1% non-essential amino acids (NEAA, Sigma Chemical Co., St. Louis, MO, USA). PLC/PRF/5 cells were cultured in DMEM (1g/L glucose) containing 10% fetal bovine serum and 1% penicillin/streptomycin (Sigma Chemical Co., St. Louis, MO, USA) but no NEAA. Both cell lines were incubated at 37°C in a humid incubator with 5% CO_2_.

### Measurement of human AFP in cell supernatant

The levels of human AFP in cell supernatants was evaluated using an ELISA kit from HUMAN (GmbH Germany) as recommended by the manufacturer. Briefly, HepG2/C3A and PLC/PRF/5 cells were seeded in 75-cm^2^ flasks in culture media. At 80% confluence, the cells were treated as described in the figure legends. The supernatant was then collected and diluted 1/50 with serum-free media for ELISA; however, the supernatants from PLC/PRF/5 were not diluted. The ELISA method is based on the affinity of biotin for streptavidin fixed on the surface of a microtiter well. The enzyme-antibody conjugate is mixed with the samples and calibrators to form the sandwich complex. After incubation and washing, the substrate is added, and a color develops. The intensity of the color is directly proportional to the concentration of AFP in the samples. The optical density (O.D.) of this colored product is measured using an ELISA reader at 450 nm.

### Measurement of human AFP in serum mice

Blood was obtained from mice tail in sterile conditions then centrifuged at 2600 rpm and 10°C for 10 min. Serum was then diluted as described in the figure legends, and ELISA (HUMAN GmbH, Germany) was performed as previously described.

### Cell apoptosis—Annexin V affinity assay

The apoptosis of HepG2/C3A and PLC/PRF/5 cells before and after treatment with telomerase inhibitors was determined using an Annexin V-FITC Apoptosis Detection Kit (Abcam) as recommended by the manufacturer. Briefly, the cells were treated with costunolide and BIBR1532 at different concentrations for 48 h. The HepG2/C3A and PLC/PRF/5 cells were then labeled with Annexin V-FITC and detected by flow cytometry.

*Transfection with siRNA of the hTERT subunit*—The HepG2/C3A and PLC/PRF/5 cell lines were seeded in 96-well plates at a density of 2x10^4^ cells/well. Before seeding, 20 nM specific siRNA, Hi-perfect (Qiagen Inc.) and serum-free medium was added to each well, and the plate was incubated for 10 min at room temperature. After incubation, the cells were added, and the plate was incubated for 72 h. TERT siRNA that targets a specific mRNA sequence of the catalytic subunit hTERT (5'-CTGGAGCAAGTTGCAAAGCAT-3) was used in this study. The sequences of the control siRNA are as follows: hTERT sense, 5'-GGAGCAAGUUGCAAAGCAUTT-3; antisense, 5'-AUGCUUUGCAACUUGCUCCAG-3 '. Transfection with siRNAs was performed using the RNAi Human / Mouse Starter Kit (Qiagen Inc., Valencia, CA, USA) according to the manufacturer’s recommended protocol.

*Proliferation assay*—The cell viability and proliferation was determined using the 3-(4,5-dimethylthiazol-2-yl)-5-(3-carboxymethoxyphenyl)-2-(4-sulfophenyl)-2H-tetrazolium salt assay as reported by the manufacturer’s instructions (Takara Bio Inc, Ostu, Shiga, Japan). The tetrazolium salt is cleaved into formazan by succinate-tetrazolium reductase, an enzyme that exists only in the mitochondrial respiratory chain and is active only in viable cells. The formazan production is proportional to the number of living cells in the culture. Briefly, the HepG2/C3A and PLC/PRF/5 cells were seeded at 10^4^ cells per well in 96-well plates. At 70% confluence, the cells were treated with BIBR1532 or costunolide for 48 h. After treatment, 10 μL of tetrazolium salt was added to 100 μL of culture media, and the plate was incubated for 45 min. The absorbance was then measured using an ELISA reader at 450 nm.

### RNA purification and reverse transcription polymerase chain reaction (RT-PCR)

The total RNA from HepG2/C3A and PLC/PRF/5 cells was isolated using the GenElute Mammalian Total RNA Kit from Sigma-Aldrich (USA) according to the manufacturer’s instructions. cDNA was synthesized from 1 ug of RNA using the iScript cDNA Synthesis Kit (Bio-Rad, USA). Quantitative PCR was performed using the REDTaq Ready Mix PCR Reaction Mix kit (Sigma-Aldrich, USA). The PCR primers sequences were as follows: AFP forward, 5’-ACCCTGGTGTTGGCCAGTGC-3’; AFP reverse, 5’-GCAGCGCTACACCCTGAGCT-3’; GAPDH forward, 5’TGGGATGGACTGTGGTCATGAG-3’; GAPDH reverse, 5’-ACTGGCGTCTTCACCACCATGG-3’. The amplified DNA was then run in a 2% agarose gel and visualized by SYBR Safe staining using the UVP BioDoc system (UVP).

### Migration assay

The assay of HepG2/C3A and PLC/PRF/5 cell migration was performed using a Boyden chamber in a 24-well plate designed by CellBiolabs (USA). According to the manufacturer's recommendations, 10^6^ cells in DMEM without serum were suspended in the upper chamber of each well. The same medium supplemented with 10% serum was added to the lower chamber of each well as a chemo-attractant solution. After 24 h, the cells that migrated to the lower chamber of the wells were stained using a staining solution that fixes and stains the cell walls and cytoplasmic membranes of any cell remaining on the insert. The stain is instantly dissolved once the kit extraction solution is added. The solution was then transferred to a 96-well microtiter plate, and the absorbance was measured at 560 nm using a plate reader.

### Invasion assay

The invasion ability of the HepG2/C3A and PLC/PRF/5 cells was assayed using a Boyden chamber in a 24-well plate designed by CellBiolabs (USA). According to the manufacturer's recommendations, 10^6^ cells in DMEM without serum were suspended in the upper chamber of each well. The same medium supplemented with 10% serum was added to the lower chamber of each well as a chemo-attractant solution. After 48 h, the cells that invaded the bottom of the membrane were stained. An extraction solution was then added, and the mixture was transferred to a 96-well microtiter plate. The cells were then quantified at 560 nm using a plate reader.

### *In vivo* experiments

Seven- to eleven-week-old male NSG mice were inoculated subcutaneously with 10^7^ HepG_2_/C_3_A cells into the right flank. Twelve days after injection, the mice were divided into a control (n = 10) and a treated (n = 10) group. The survival of the mice was monitored every day. The animals received sterile food and water. The animals in the treated group were injected intraperitoneally with 30 mg/kg costunolide, whereas the mice in the control group received the vehicle. The injections were performed for six successive days. Blood was drawn from the tail vein for AFP quantification as described in the results. The tumor size was measured using a digital caliper every five days, and the volumes were estimated using the following formula: 0.5 × *length* × (*width*)2 [57]. This study was approved by the Ethics Committee of Faculty of Medicine of Saint Joseph’s University.

*Statistical analysis*—The results were assessed by *t*-tests using the GraphPad QuickCalcs online software (http://www.graphpad.com/quickcalcs/ttest1.cfm). The values are expressed as the means ± SD.

## Results

### Effect of telomerase inhibitors on AFP expression and secretion

**Fig 1:**
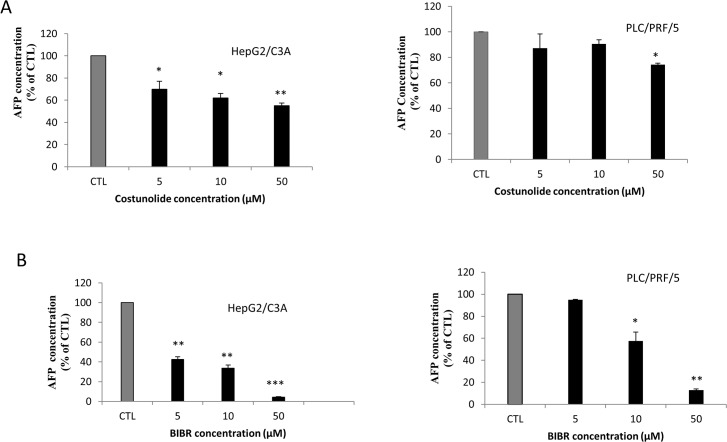
Effect of costunolide and BIBR1532 on AFP secretion by HepG2/C3A and PLC/PRF/5 cell lines. The cells were seeded in 75-cm^2^ flasks in DMEM (1g/L). At 80% confluence, the cells were treated with costunolide (5, 10 or 50 μM) or BIBR1532 (5, 10 or 50 μM) for 48 h. The supernatant was then collected for ELISA. (A) Costunolide (5–50 μM) significantly decreased AFP production in a dose-dependent manner in the supernatants of HepG2/C3A cells after 48 h of treatment, whereas 50 μM costunolide decreased the AFP levels in the supernatant of PLC/PRF/5 cells. (B) BIBR1532 (5, 10 or 50 μM) significantly decreased AFP production in a dose-dependent manner in the supernatants of HepG2/C3A cells after 48 h of treatment. The same pattern was observed in the supernatants of PLC/PRF/5 cells after treatment with BIBR1532 at 10 or 50 μM. Each value represents the mean of at least three different assays. Each value had its own control which was set as 100%. Each datapoint represents the mean ± SD from all of the experiments. *P<0.01 versus untreated cells. **P<0.005 versus untreated cells.

**Fig 2:**
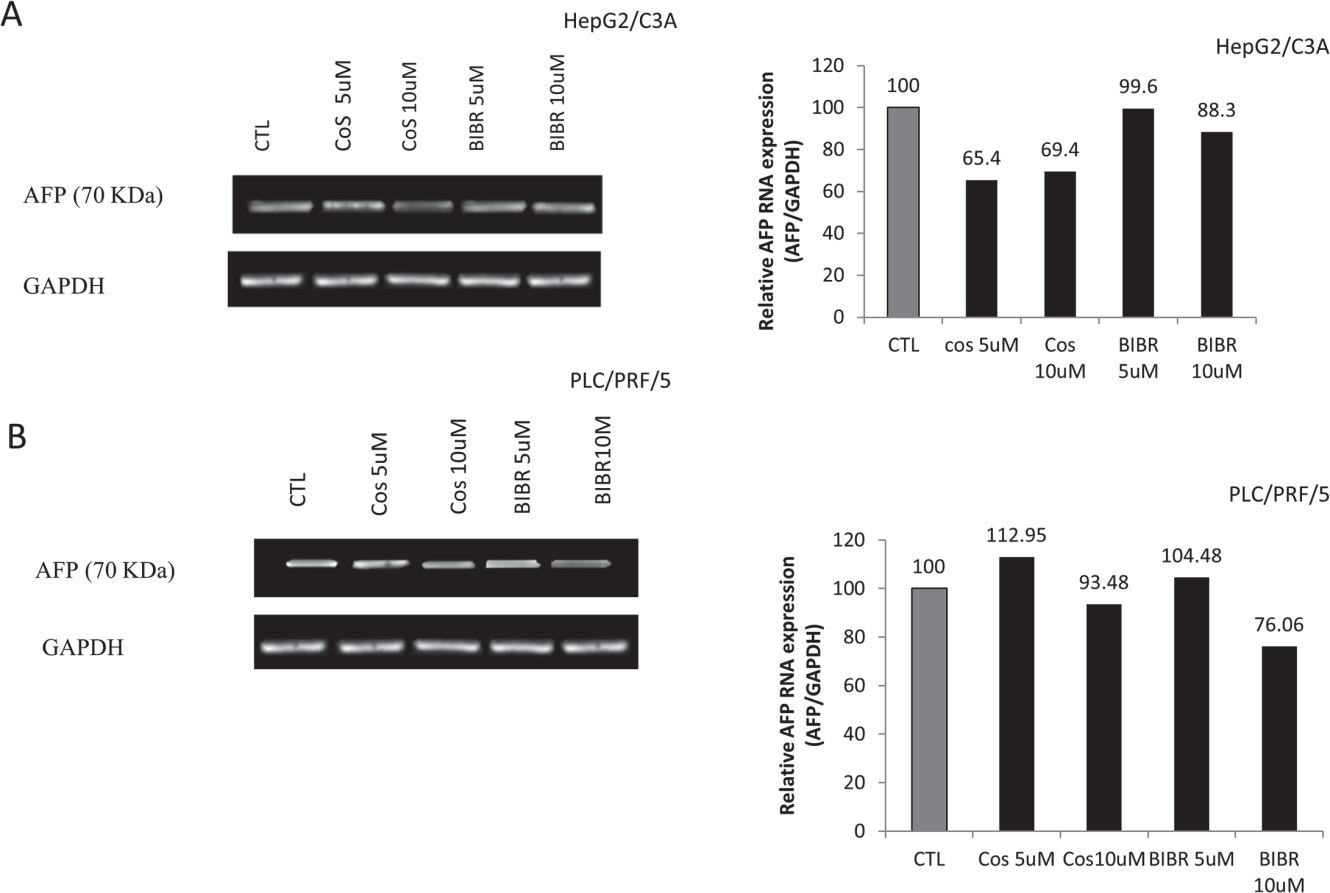
Effect of costunolide and BIBR1532 on APF mRNA expression by HepG2/C3A and PLC/PRF/5 cell lines. The cells were seeded in 75-cm^2^ flasks in DMEM (1g/L). At 80% confluence, the cells were treated with costunolide (5, 10 or 50 μM) or BIBR1532 (5, 10 or 50 μM) for 48 h. The RNA from the cells was then extracted for RT-PCR. The amplified DNA was then run in a 2% agarose gel and visualized with SYBR Safe staining. The relative mRNA levels were obtained using the Gel Analyzer software. (A) Semi-quantitative reverse transcription- PCR analysis of AFP mRNA in HepG2/C3A cells after treatment with costunolide and BIBR1532 (5 and 10 μM). Costunolide (10 μM) and BIBR1532 (10 μM) decreased the AFP mRNA levels by 30.6% and 11.7%, respectively. (B) Costunolide had no significant effect on AFP mRNA expression in PLC/PRF/5 cells, whereas BIBR1532 (10 μM) decreased the AFP mRNA level by 23.94%. Each experiment was repeated at least three different times.

To investigate the role of telomerase inhibition on AFP secretion, we treated cells belonging to the hepatocellular carcinoma cell lines HepG2/C3A and PLC/PRF/5 with two different telomerase inhibitors, namely costunolide and BIBR1532. The results, which are shown in “Fig. 1”, reveal the effect of telomerase inhibition on AFP secretion. In fact, costunolide dose-dependently (5–50 μM); “Fig. 1A”; decreases AFP secretion (25–40%) by HepG2/C3A cells. The same pattern was observed “Fig. 1B” with BIBR 1532 (5–100 μM), but the inhibition was more pronounced (60–100%). The treatment of PLC/PRF/5 “Fig. 1A” cells with costunolide at concentrations of 5 or 10 μM exerted no significant effects, whereas at 50 μM, the AFP concentration in the cell supernatants decreased by 30%. Furthermore, the treatment of the cells with BIBR1532 at 10 or 50 μM decreased AFP secretion by 60–80% “Fig. 1B”. It is important to mention that costunolide inhibits the expression of hTERT, whereas BIBR1532 directly inhibits the same subunit of telomerase, which could explain the prominent effect of BIBR1532. Moreover, the effect of telomerase inhibitors was also assessed based on the mRNA expression of AFP. Both costunolide and BIBR1532 partly inhibited AFP mRNA expression in HepG2/C3A to equal extents “Fig. 2A”. In addition, treatment with 10 μM BIBR1532 also decreased the AFP mRNA expression in PLC/PRF/5 cells, whereas costunolide had no effect at concentrations of 5 or 10 μM “Fig. 2B”.

### Effect of hTERT siRNA on AFP secretion

**Fig 3:**
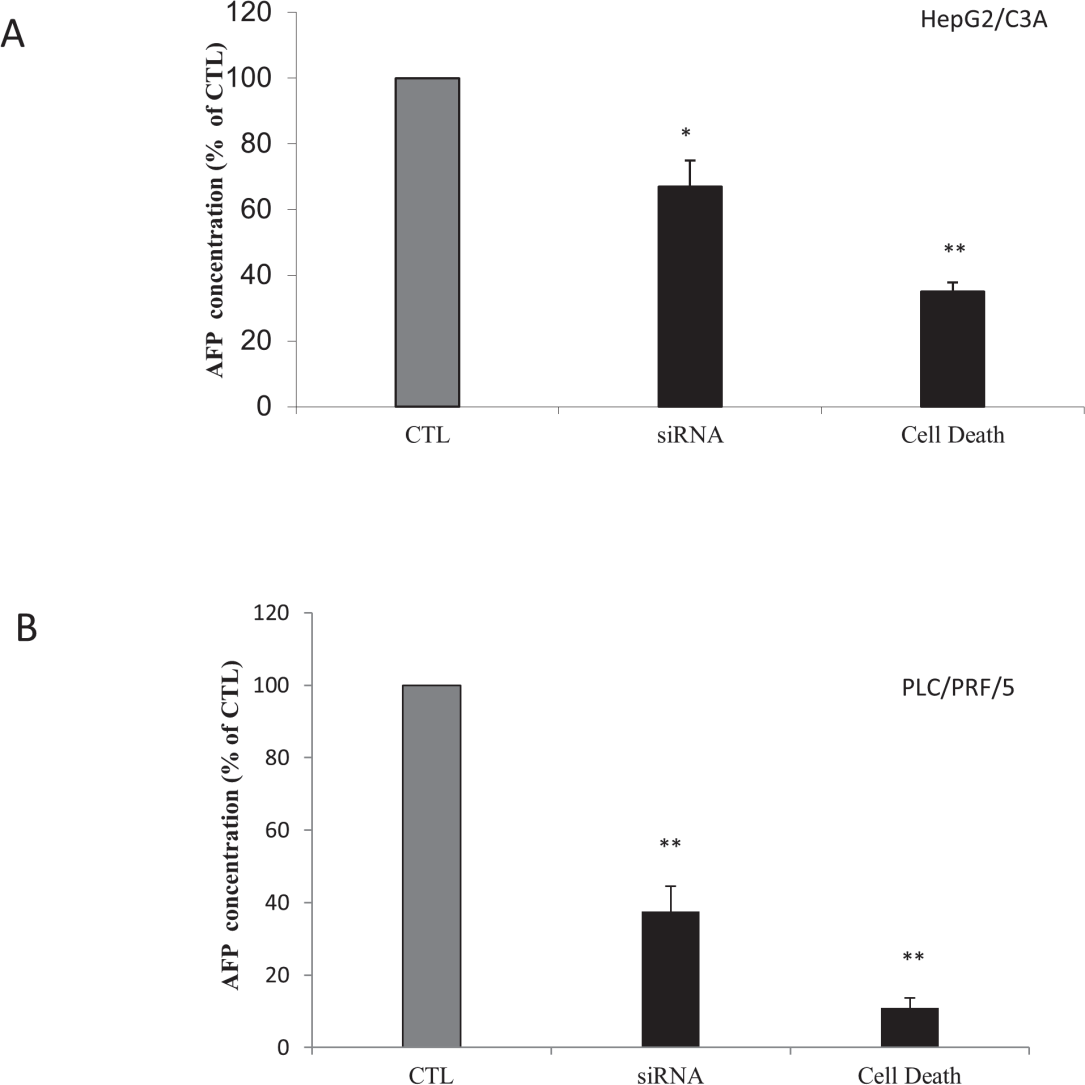
Effect of telomerase siRNA transfection on AFP production by HepG2/C3A and PLC/PRF/5 cells. The cells were seeded in 96-well plates in 100 μL of DMEM with FBS at a density of 2x10^4^ cells/well. Before seeding, the cells were treated with TransFectin and 20 nM specific siRNA or a mix of siRNAs vital for cell survival. The control received the vehicle. After incubation, 50 μL of the supernatant was collected and tested by ELISA for AFP quantification. (A) The inhibition of telomerase by hTERT siRNA induced a decrease in AFP production by HepG2/C3A cells. (B) The same pattern was observed in the supernatants of PLC/PRF/5 cells. Each value was normalized to its own control, which was set to 100%. The data represent the means ± SD from three different experiments. *P<0.01 versus untransfected cells. **P<0.005 versus untransfected cells.

To confirm the direct involvement of telomerase in AFP expression and secretion modulation and to exclude any extratelomeric action of its inhibitors, namely costunolide and BIBR 1532, we treated HepG2/C3A and PLC/PRF/5 cells with hTERT siRNA “Fig. 3” and with a blend of siRNAs that silence several proteins essential for cell survival, which was used as a positive control for transfection success. As expected, the pattern obtained with HepG2/C3A cells presented similar inhibition of AFP secretion as that obtained with the telomerase inhibitors. However, 25 nM hTERT siRNA inhibited AFP secretion by approximately 40%, which is similar to that for HepG2/C3A cells treated with 10 μM costunolide and for PLC/PRF/5 cells treated with 10 μM BIBR1532.

### Effect of telomerase inhibitors on cell migration

**Fig 4:**
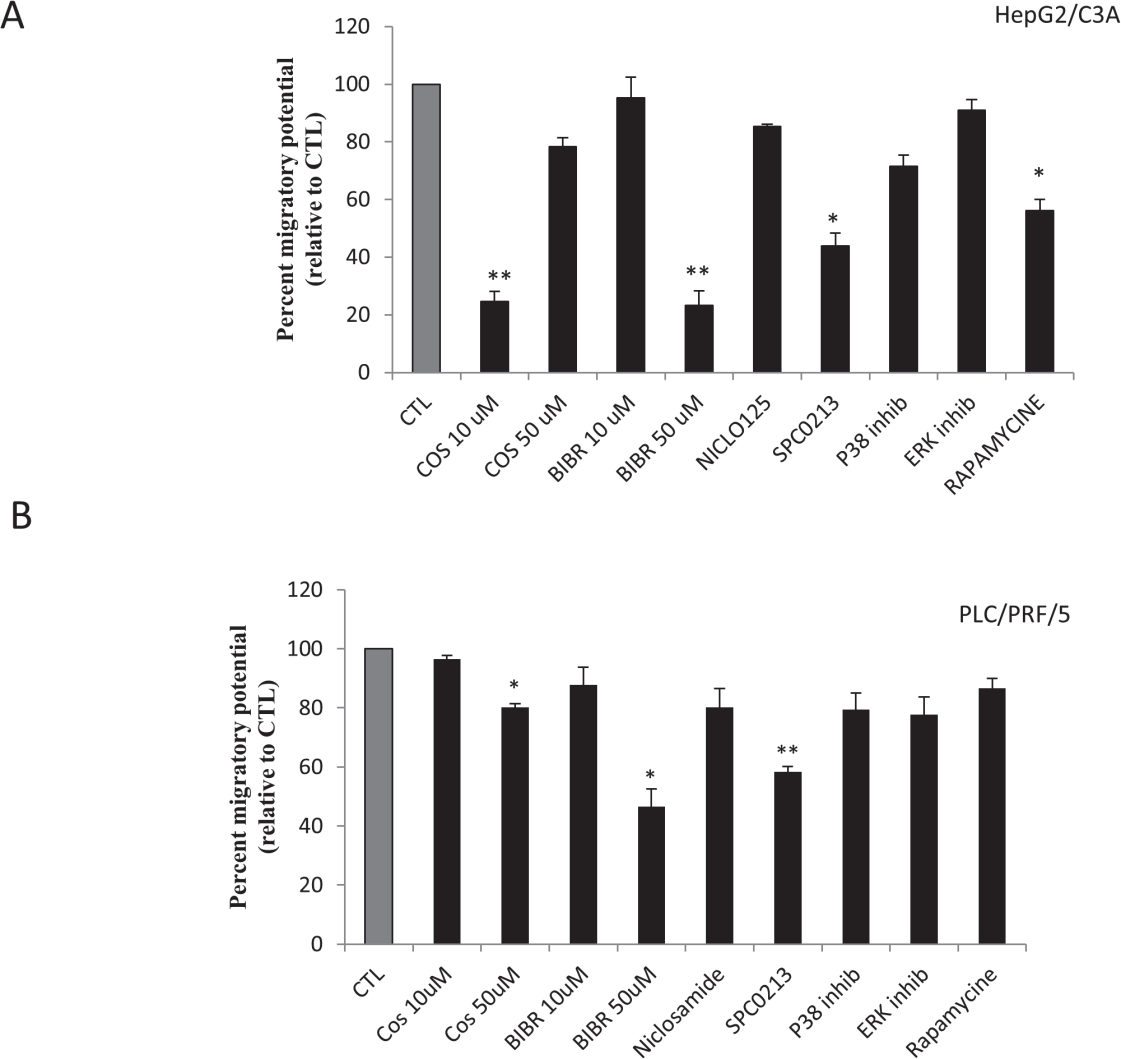
Effect of telomerase, mTOR and PKC inhibitors on HepG2/C3A and PLC/PRF/5 cell migration. The migration of HepG2/C3A and **PLC/PRF/5** cells was assayed using the Boyden chamber technique in a 24-well plate. According to the manufacturer’s recommendations, 10^6^ cells in DMEM without serum were suspended in the upper chamber of each well; in the lower chamber, DMEM + 10% FBS was added as a chemo-attractant solution. After 24 h of treatment, the cells that migrated to the lower chamber were stained using a staining solution that fixes and stains the cell walls and cytoplasmic membranes of any cell remaining on the insert. The stain is instantly dissolved once the kit extraction solution is added. The solution was then transferred to a 96-well microtiter plate, and the absorbance was measured at 560 nm using a plate reader. (A) Costunolide (10 μM), BIBR (10 μM), rapamycin (200 nM) and SPC0213 (5 μM) had a significant effect on HepG2/C3A cell migration after treatment for 24 h. (B) Costunolide (50 μM), BIBR (50 μM) and SPC0213 (5 μM) had a significant effect on PLC/PRF/5 cell migration after treatment for 24 h. Each value represents the mean of three assays. Each value was normalized to its own control, which was set to 100%. Each point represents the mean ± SD from all of the experiments. *P<0.01 versus untreated cells. **P<0.005 versus untreated cells.

Cell migration is one of several hallmarks of cancer. “Fig. 4” shows the effect of telomerase inhibition on HepG2/C3A and PLC/PRF/5 cell migration. As clearly shown, both costunolide at 10 μM and BIBR 1532 at 10 μM significantly decreased HepG2/C3A cell migration, as assessed by the Boyden chamber technique. Similar results were obtained with mTOR and PKC inhibition by 200 nM rapamycin and 5 μM SPC0213, respectively “Fig. 4A”. The same pattern was obtained for PLC/PRF/5 cells treated with a PKC inhibitor (5 μM), but a high concentration of telomerase inhibitors (50 μM) was required to decrease AFP secretion by PLC/PRF/5 cells “Fig. 4B”.

### Effect of telomerase inhibition on cell invasion

**Fig 5:**
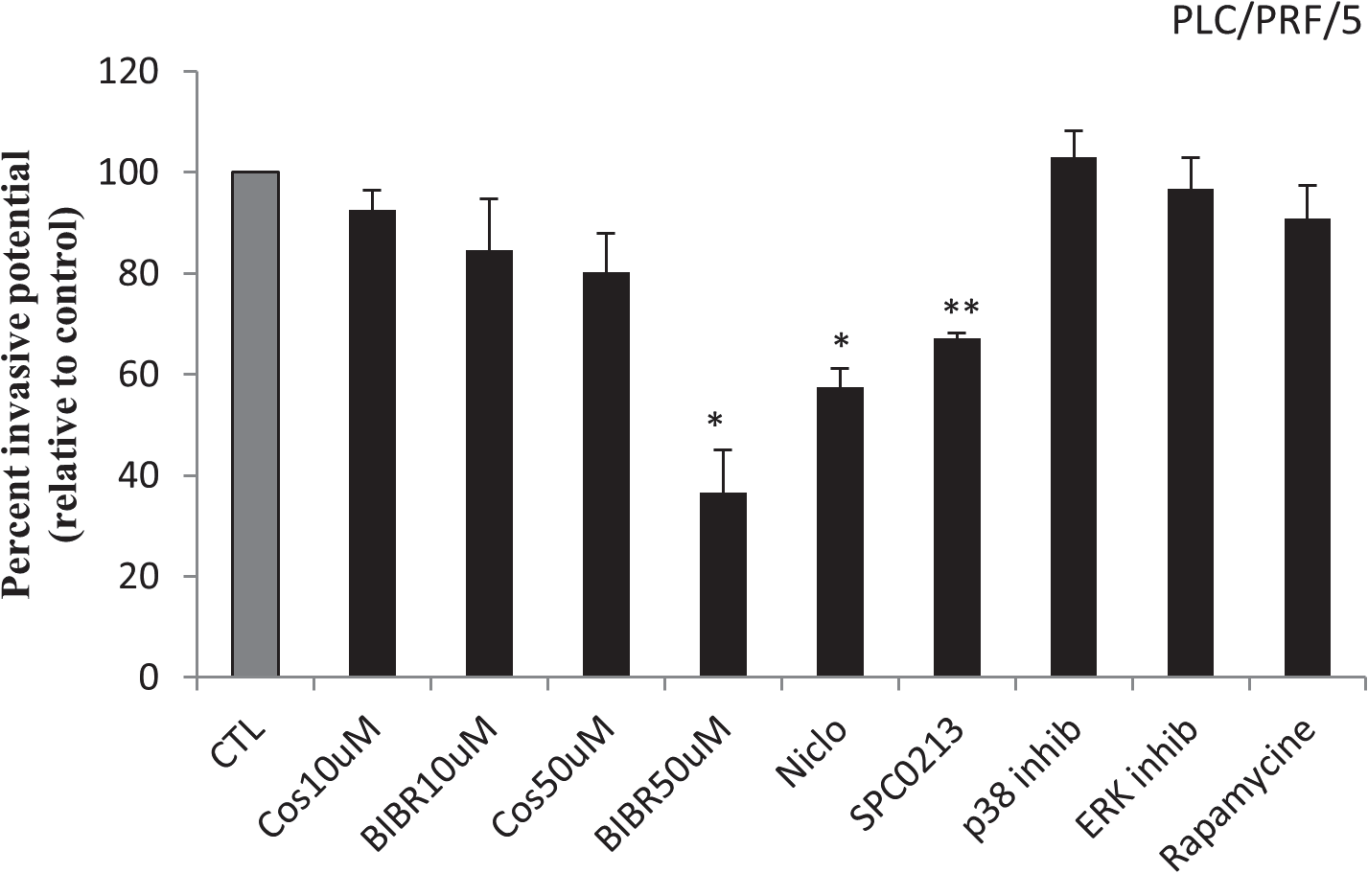
Effect of telomerase, mTOR and PKC inhibitors on PLC/PRF/5 cell invasion. The invasion of PLC/PRF/5 cells was assayed using the Boyden chamber technique in a 24-well plate. According to the manufacturer’s recommendations, 10^6^ cells in DMEM without serum were suspended in the upper chamber of each well. The same medium supplemented with 10% serum was added to the lower chamber of each well as a chemo-attractant solution. After 48 h, the cells that invaded the bottom of the membrane were stained. An extraction solution was then added, and the mixture was transferred to a 96-well microtiter plate. The cells were quantified at 560 nm using a plate reader. BIBR1532 (50 μM), niclosamide (125 nM) and SPC0213 (5 μM) had a significant effect on PLC/PRF/5 invasion. Each value represents the mean of three assays. Each value was normalized to its own control, which was set to 100%. Each point represents the mean ± SD from all of the experiments. *P<0.01 versus untreated cells. **P<0.005 versus untreated cells.

An *in vitro* invasion assay was performed to determine the metastatic potential of HepG2/C3A and PLC/PRF/5 cells after treatment with telomerase, STAT3 and PKC inhibitors. As shown in “Fig. 5”, BIBR1532 (50 μM) and niclosamide (125 nM) inhibited PLC/PRF/5 cell invasion compared with untreated control cells, whereas the inhibition of PKC by SPC0213 (5 μM) had a more significant effect on cell invasion (p < 0.01). Although, the HepG2/C3A cells are known to be non invasive, the invasion assay was also performed on these cells. Treated and untreated cells did not show any invasive properties (data not shown).

### Effect of telomerase inhibitors on cells apoptosis

To exclude any apoptotic effect of telomerase inhibitors that may contribute to the decreased AFP secretion and/or expression and thus provide false-negative results, we measured the apoptosis of HepG2/C3A and PLC/PRF/5 cells by the detection of Annexin using a flow cytometry technique. The results of three independent experiments are reported in “Table 1” and clearly show that neither costunolide nor BIBR1532 at low or high concentration exerted an apoptotic effect that may contribute to cell death.

**Table 1:** Annexin V sorting of HepG2/C3A and PLC/PRF/5.

	HepG2/C3Acells				PLCcells			
	Cos5μM	Cos10μM	BIBR5μM	BIBR10μM	Cos5μM	Cos10μM	BIBR5μM	BIBR10μM
AnnexinV+(%)	0.38	1.98	0.22	0.76	2.29	0.4	0.96	0.17
AnnexinV-(%)	0.25	1.56	0.35	0.65	3.05	0.29	0.38	0.17

HepG2/C3A and PLC/PRF/5 were seeded in a 24-well plates at a density of 10^5^ cells/well. At 80% confluence, the cells were treated with costunolide (5 or 10 μM) or BIBR1532 (5 or 10 μM) for 48 h. Annexin V assay was then performed using a flow cytometer. The results are displayed as percentage of control.

### *In vivo* study: effect of costunolide on AFP secretion in NSG mice

The encouraging *in vitro* results prompted us to further investigate the effect of telomerase inhibitors *in vivo*. Because HepG2/C3A cells express and secrete more AFP than PLC/PRF/5 cells and because HepG2/C3A cells exhibited a more pronounced response to treatment with telomerase inhibitors, we decided to use this cell line in our *in vivo* study.

**Fig 6:**
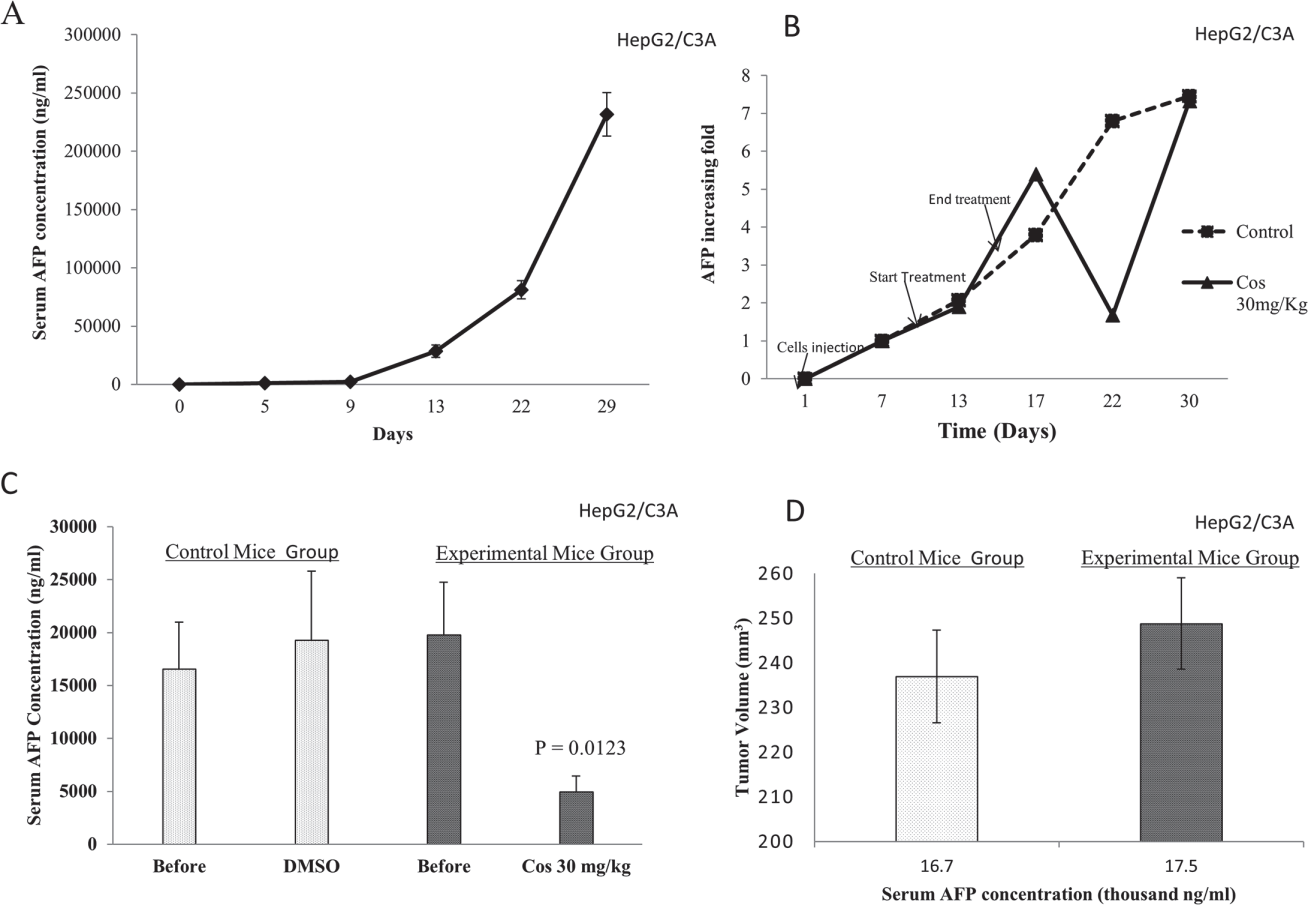
In *vivo* effect of costunolide on AFP secretion in NSG mice. NSG mice were inoculated with HepG2/C3A cells and treated as described in Materials and Methods. (A) AFP kinetics in the mice blood after cell injection. (B) A representative figure of the effect of costunolide 30 mg/kg on AFP secretion in the NSG mice blood. (C) Mean and standard error of AFP secretion in the NSG mice blood before and after treatment with 30 mg/kg costunolide. The control group received the vehicle. (D) Mean tumor size as a function of the AFP secretion in the NSG mice blood before and after treatment.

The immunodeficient NSG mice (also known as SCID/NOD) were used for this *in vivo* study. Our first experiment aimed to assess the secretion of human AFP in the mice serum after injection of the cells, as described in the Materials and Methods. “Fig. 6A” shows that AFP was substantially detected after five days (∼1118 ng/ml), and the AFP kinetics were then monitored for 29 days, which revealed that the AFP concentration increased in an exponential manner to reach approximately 230,000 ng/mL. We then investigated the effect of costunolide at 30 mg/kg; as demonstrated *in vitro*, the *in vivo* results “Fig. 6B” clearly show that costunolide decreased the serum AFP concentration in mice by six-fold compared with the control group. Surprisingly, the effect of costunolide was achieved several days after the costunolide injection. In fact, the mice started receiving the injections on day 12 for six consecutive days, and at day 17, the injections were stopped. On day 17, the AFP concentration was slightly elevated compared with that obtained for the control group. Significantly decreased AFP secretion was obtained on day 22, at which the concentration was markedly decreased six-fold compared with that obtained for the control group, which received the vehicle. “Fig. 6B” presents the results from one experiment, whereas “Fig. 6C” shows the average from at least 20 experimental and control mice. After treatment, the AFP concentration decreased by 75% compared with the control group. However, no differences in the mice tumor sizes were noted between control and experimental groups “Fig. 6D”.

### Role of the PI3K/Akt/mTOR signaling pathway

**Fig 7:**
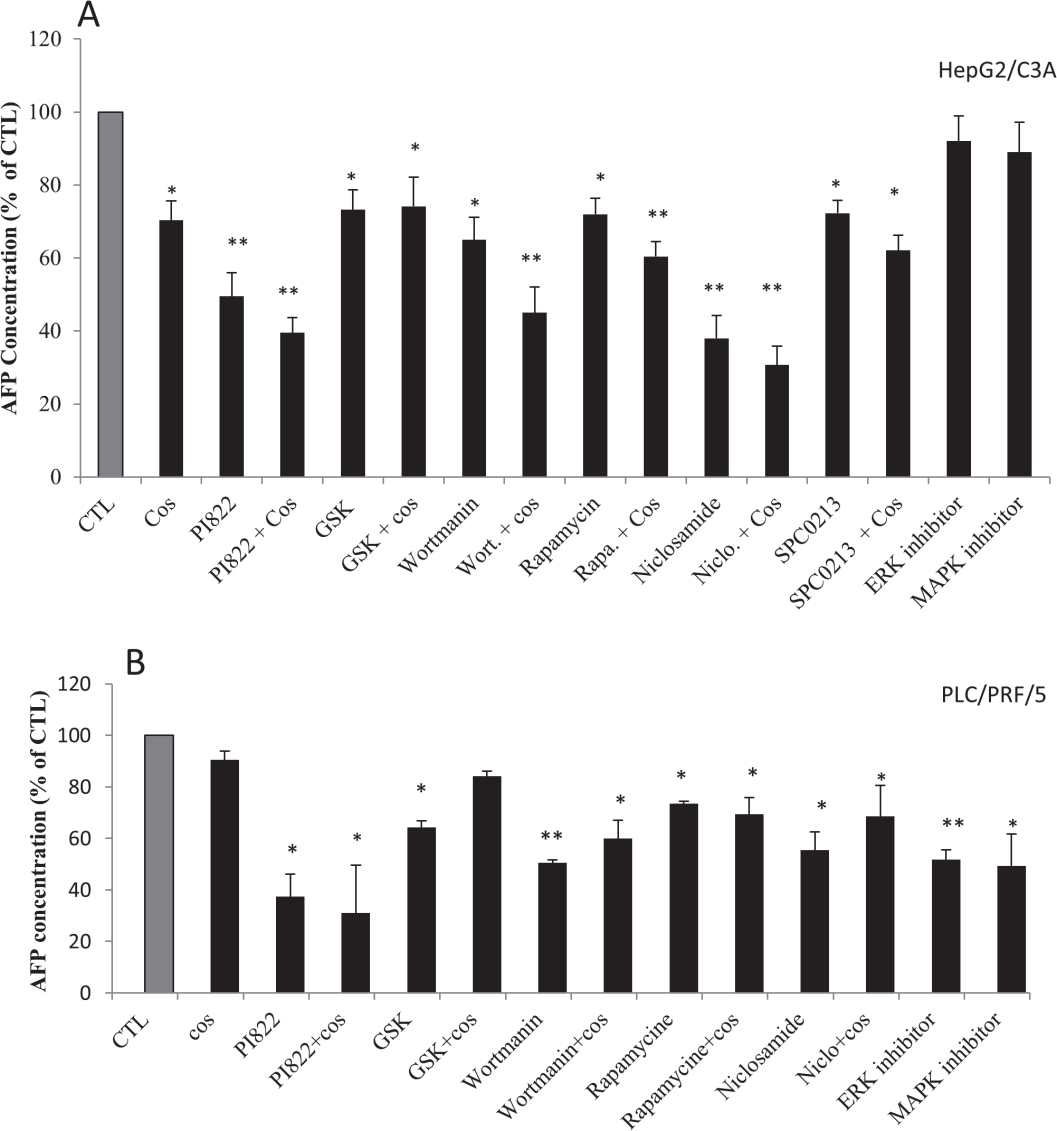
Effect of pathway inhibitors in the presence or absence of costunolide on AFP expression and secretion. The cells were seeded in a six-well plate at a density of 2x10^5^ cells/well. At 80% confluence, the cells were treated with inhibitors of the PI3k/mTOR/Akt and MAPK/ERK signaling pathways and costunolide for 48 h. The supernatants were then collected and assessed by ELISA. (A) Effect of inhibitors of the PI3K/Akt/mTOR pathway on AFP secretion by HepG2/C3A cells. (B) Effect of inhibitors of the PI3K/Akt/mTOR pathway on AFP secretion by PLC/PRF/5 cells. *P<0.05 versus untreated cells. **P<0.01 versus untreated cells.

**Fig 8:**
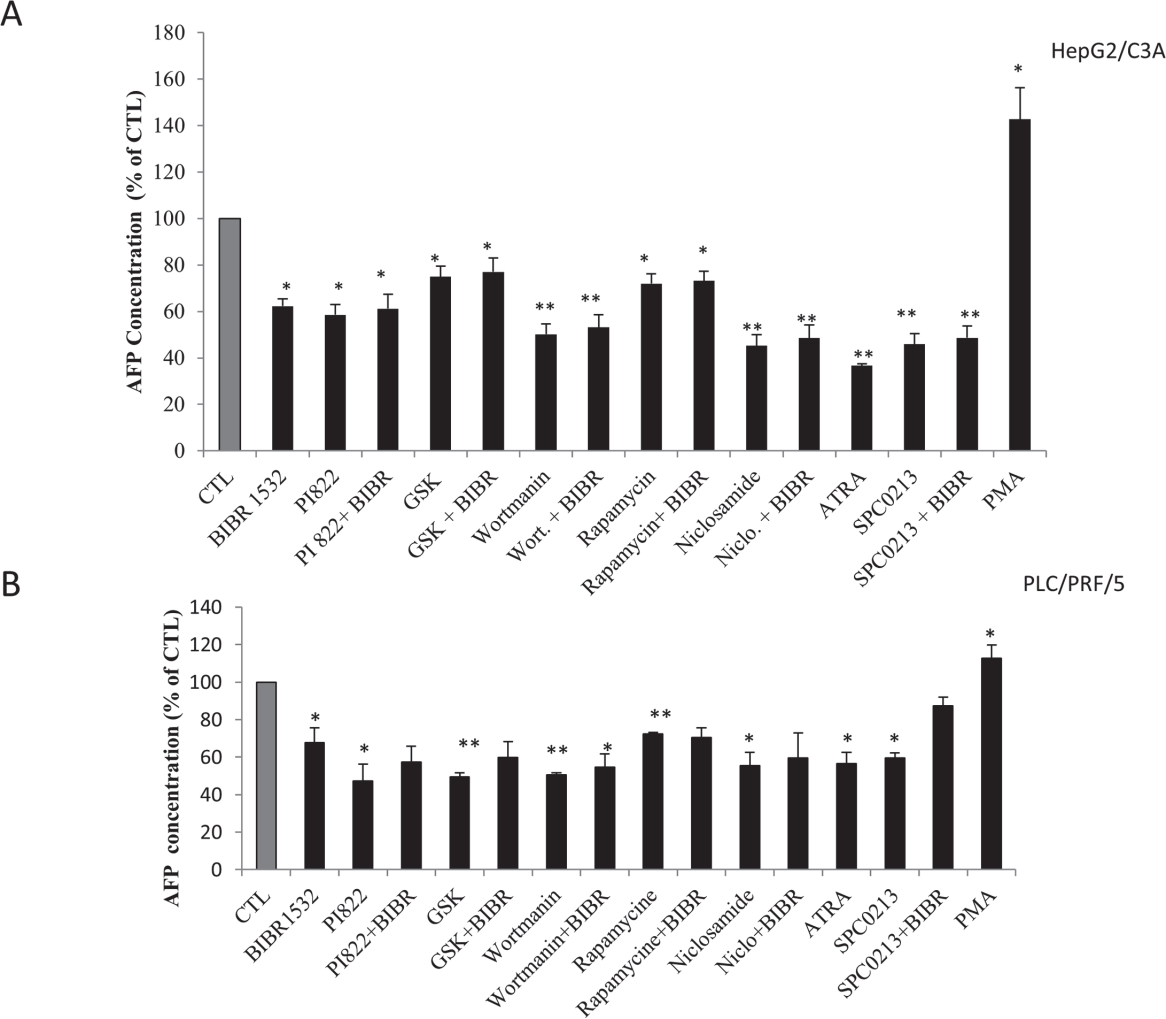
Effect of pathway inhibitors in the presence or absence of BIBR1532 on AFP expression and secretion. The cells were seeded in a six-well plate at a density of 2x10^5^ cells/well. At 80% confluence, the cells were treated with inhibitors of the PI3k/mTOR/Akt and MAPK/ERK pathways and BIBR1532 for 48 h. The supernatants were then collected and evaluated by ELISA. (A) Effect of inhibitors of the PI3K/Akt/mTOR pathway on AFP secretion by HepG2/C3A cells. (B) Effect of inhibitors of the PI3K/Akt/mTOR pathway on AFP secretion by PLC/PRF/5 cells. *P<0.05 versus untreated cells. **P<0.01 versus untreated cells.

**Fig 9:**
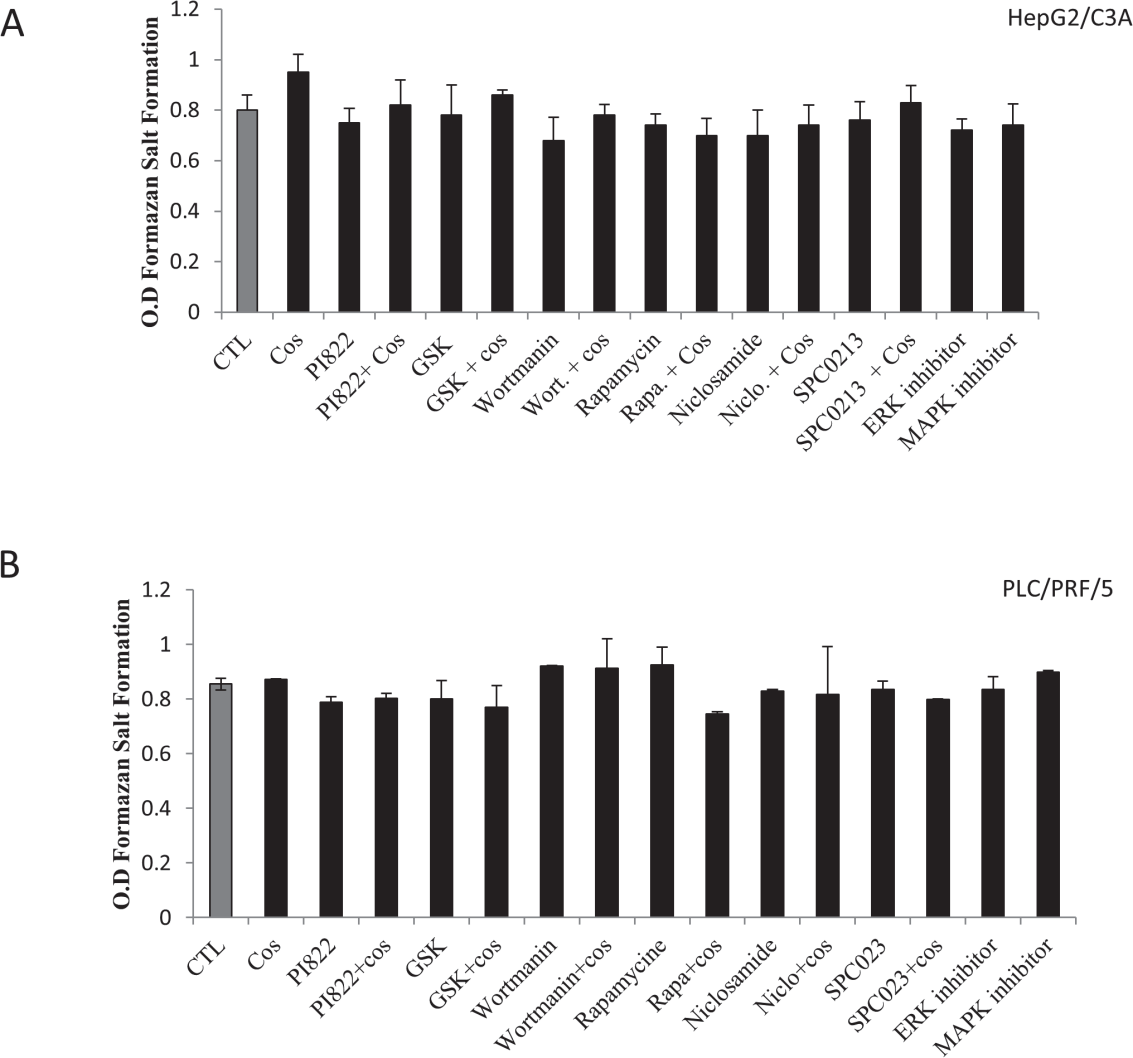
Effect of pathway inhibitors in the presence or absence of costunolide on HepG2/C3A and PLC/PRF/5 cell proliferation. The cells were seeded in a 96-well plate at a density of 10^4^ cells/well. At 80% confluence, the cells were treated with inhibitors of the PI3k/mTOR/Akt and MAPK/ERK pathways and costunolide for 48 h. After treatment, 10 μL of tetrazolium salt was added to 100 μL of the culture media, and the mixture was incubated for 45 min. The absorbance was then measured using an ELISA reader at 450 nm. As shown in “Figs A and B”, the inhibition of PI3K/Akt/mTOR and PKC had no significant effect on the proliferation of cells incubated in the presence or absence of costunolide.

**Fig 10:**
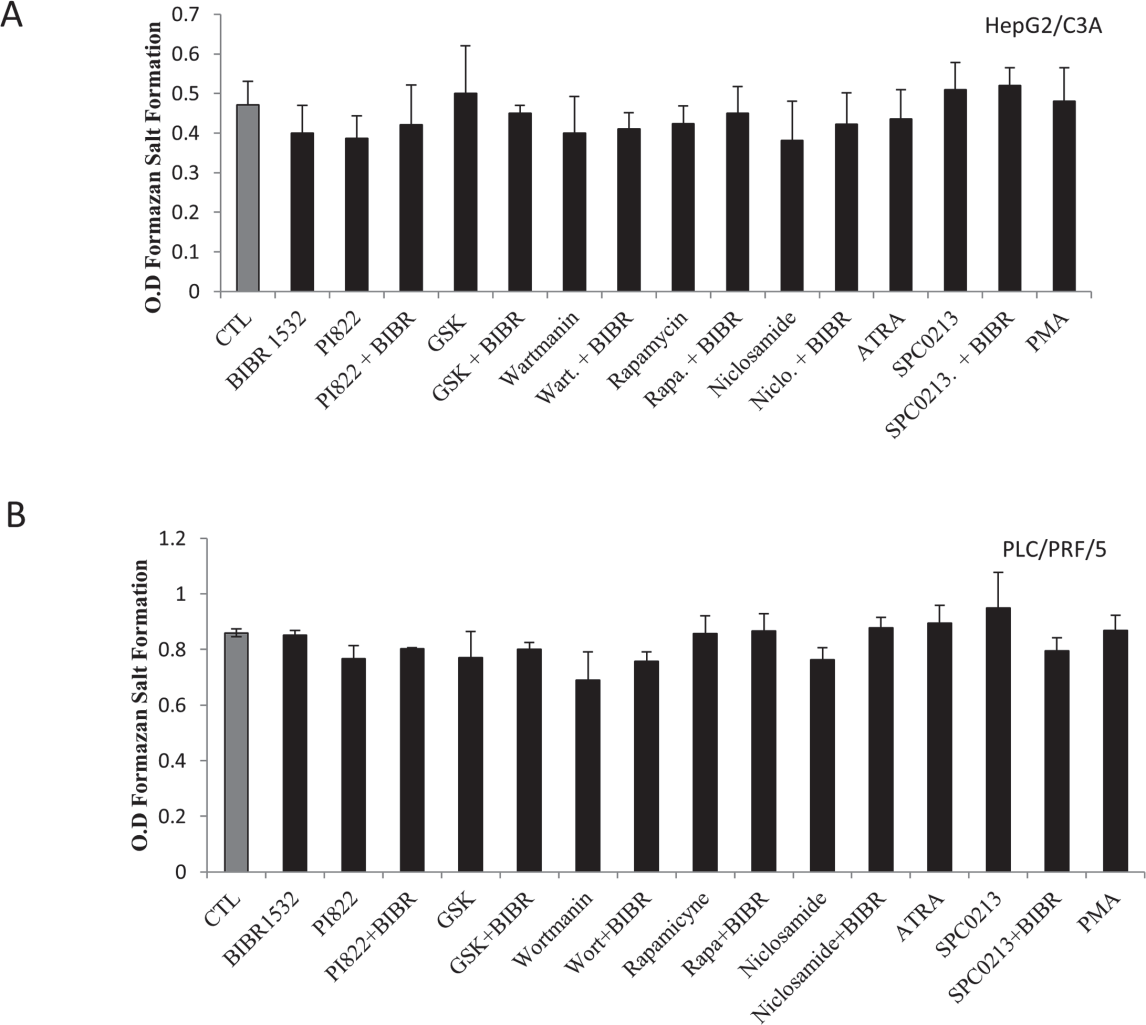
Effect of pathway inhibitors in the presence or absence of BIBR1532 on HepG2/C3A and PLC/PRF/5 cell proliferation. The cells were seeded in a six-well plate at a density of 10^4^ cells/well. At 80% confluence, the cells were treated with inhibitors of the PI3k/mTOR/Akt and MAPK/ERK pathways and BIBR1532 for 48 h. After treatment, 10 μL of tetrazolium salt was added to 100 μL of the culture media, and the mixture was incubated for 45 min. The absorbance was then measured using an ELISA reader at 450 nm. As shown in “Figs. A and B”, the inhibition of PI3K/Akt/mTOR and PKC had no significant effect on the proliferation of cells incubated in the presence or absence of BIBR1532.

**Fig 11:**
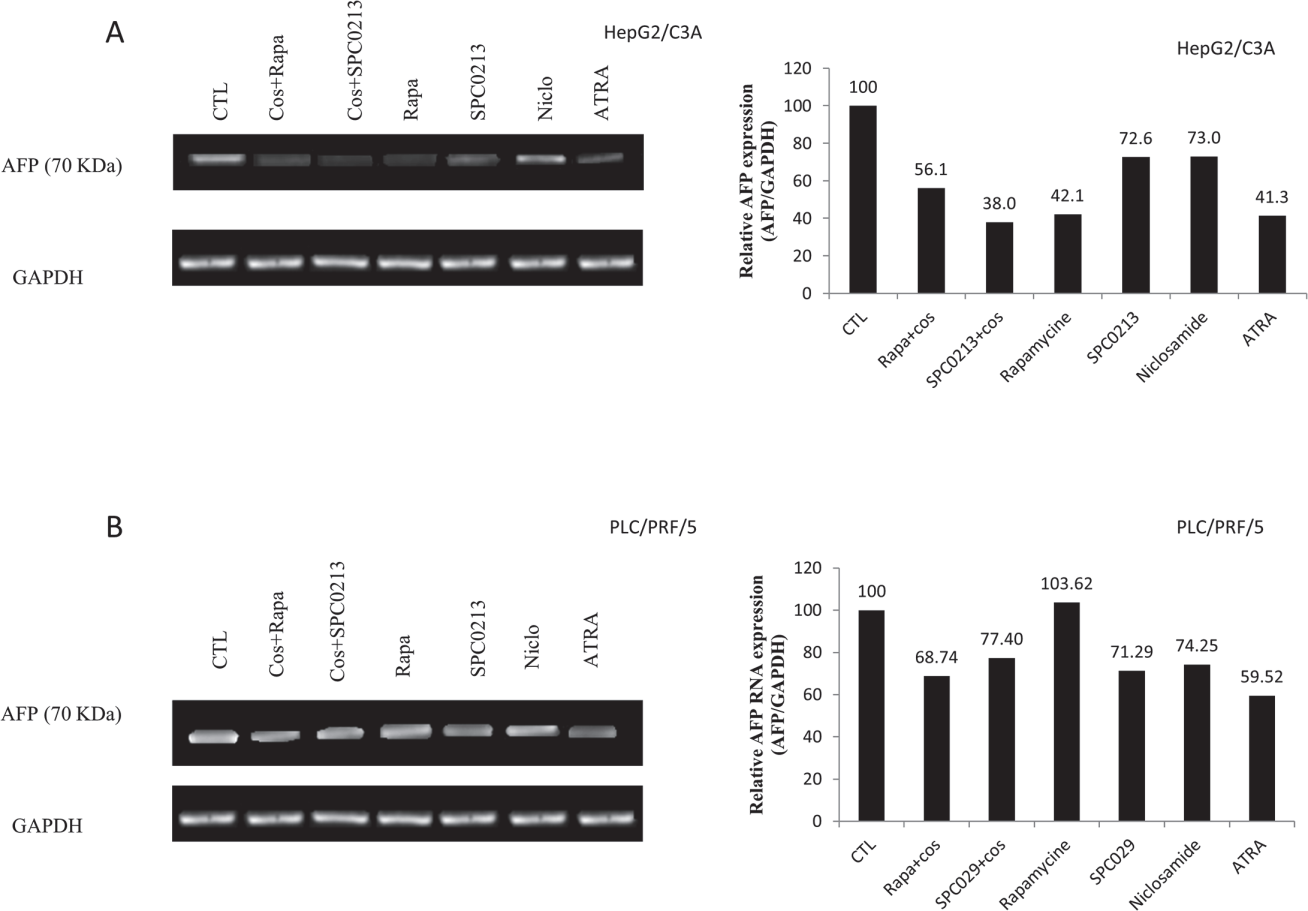
Effect of pathway inhibitors on AFP mRNA in HepG2/C3A and PLC/PRF/5 cells. The cells were seeded in a six-well plate at a density of 10^4^ cells/well. At 80% confluence, the cells were treated with inhibitors of PI3k/mTOR/Akt and costunolide for 48 h. The cell RNA was then extracted for RT-PCR, and this step was followed by cDNA amplification. The cDNA was then run in a 2% agarose gel and visualized by SYBR Safe staining. The relative mRNA levels were obtained using the Gel Analyzer software. Each experiment was repeated at least three different times.

To elucidate the signaling pathway involved in the AFP modulation, we assessed the role of the two most activated signaling pathways in cancer cells: the PI3k/Akt/mTOR and the MAPK/ERK pathways. However, “Figs. 7 and 8” show the effect of three different PI3K inhibitors, namely PI822, GSK1059615 and wortmannin, and the effect of rapamycin and niclosamide on mTOR and STAT3 inhibition, respectively. The results clearly demonstrated a significant decrease in AFP secretion following inhibition of this signaling pathway. The comparison of the above-mentioned inhibitors revealed that STAT3 inhibition appears to be more effective in HepG2/C3A cells. In addition, the combination of costunolide with the inhibitors potentiated the effect; however, it appears that this effect was additive and not synergistic. We also assessed the role of the telomerase modulator PKC and the potential role of the MAPK/ERK pathway in the modulation of AFP secretion. The same AFP downregulation was obtained after PKC inhibition, whereas the treatment of HepG2/C3A cells with P38 MAPK and ERK inhibitors had no effect “Fig. 7A”. The same pattern was obtained for the treatment of PLC/PRF/5 cells with the above-mentioned inhibitors with the exception that wortmannin was the most effective among the inhibitors used and the ERK inhibitor significantly decrease AFP secretion “Fig. 7B” (these effects were not obtained with the HepG2/C3A cells). “Fig. 8” shows exactly the same pattern of AFP secretion inhibition presented in “Fig. 7”; however, the specific telomerase inhibitor BIBR1532 was used instead of costunolide. However, the addition of BIBR1532 to the other inhibitors did not potentiate the effect of the inhibitors. The same figure also demonstrates that ATRA, which is known to decrease AFP secretion and was used as a positive control, downregulated AFP secretion into the culture media of the cells. In addition, as expected, PKC activated with PMA stimulated the secretion of AFP, as shown in the figure. The same pattern was observed in PLC/PRF/5 cells “Fig. 8B”. “Figs. 9 and 10” show the results of the viability and toxicity assays of HepG2/C3A and PLC/PRF/5 cells after treatment with the different above-mentioned inhibitors presented in “Figs. 7 and 8”. No significant toxic effect or decrease in cell viability was noted after cell treatment. In addition, “Fig. 11A” showed that the effect on AFP secretion by HepG2/C3A cells was at both the protein and posttranscriptional levels. This effect was also shown in PLC/PRF/5 cells “Fig. 11B” after treatment with costunolide, SPC0213 and niclosamide, but rapamycin had no effect on AFP mRNA secretion.

## Discussion

AFP has long been considered a marker for monitoring hepatocellular carcinoma treatment but is not reliable in some cases. The importance of AFP in clinical follow-up and treatment outcome was recently seriously investigated, as reported by three different clinical studies. In fact, the first study, which was conducted by Chan et al. [58] showed that AFP serial measurements correlate well with the prognostication and monitoring of the treatment response in patients with hepatocellular carcinoma undergoing systemic chemotherapy. These researchers recommended the use of this serum marker in routine clinical assessment of the treatment outcome and in future clinical trials because AFP is an indicator of chemotherapy efficiency. The second study [59], the results of which are supported by a study conducted by Vora et al. [60], showed that the AFP blood concentration correlates with the radiologic treatment response of patients with hepatocellular carcinoma and that these AFP changes significantly correlate with radiological changes. The third study [37] investigated the value of AFP for the measurement of the outcome in patients with hepatocellular carcinoma after locoregional therapy. The results of this study noted the important role of AFP as a reliable predictor of the radiological response, progression, and survival of these patients with HCC. Taken together, the previous studies highly suggest that AFP correlates with the aggressivity of HCC and that its serum concentration in patients predicts the treatment outcome. In addition, some studies have showed a correlation between elevated AFP serum levels and neoplasm size [61] or the degree of differentiation and etiology [62]. Combining this observation with the facts that telomerase is responsible for cell immortalization in more than 85% of cancers, including HCC, and that its mRNA is detected in the serum of patients with HCC [63], we investigated the possible interaction between telomerase and AFP. Our *in vitro* results clearly show that specific telomerase inhibition by costunolide, BIBR1532 and the hTERT siRNA decreased AFP secretion in the culture media of HepG2/C3A cells. This inhibition may have a negative effect on cancer cell proliferation because AFP is known to have a proliferative effect on cancer cells [31]. The same effect was observed *in vivo*. However, some unexpected results were observed: the decrease in AFP was delayed for five days after costunolide treatment. This delay may be due to either the time required for the mice to clear human AFP or for the genomic action of the telomerase inhibitor costunolide, which requires the modulation of several genes, as shown from the *in vitro* results that showed that costunolide and BIBR1532 decrease the expression of AFP mRNA. Treatment with costunolide did not have any effect on tumor size; however, the mice treated with costunolide looked healthier and were more active than those in the control group, even when they carried a large tumor. Several years earlier, studies on sorafenib [64,65], a small molecule that inhibits cell proliferation, angiogenesis and apoptosis, showed no correlation between tumor shrinkage and patient survival. Collectively, our findings demonstrate that telomerase inhibition decreases AFP expression and secretion by HepG2/C3A cells both *in vitro* and *in vivo*. Similar results were obtained *in vitro* using the hepatitis B-infected (HBsAg+) cell line, PLC/PRF/5. In fact, discrepancies in the level of AFP expressed by the cells and the effect of costunolide were observed. In fact, PLC/PRF/5 cells express and secrete less than 50-fold AFP compared with the levels observed in HepG2/C3A cells and do not respond to costunolide treatment. However, the inhibition of telomerase activity by BIBR-1532 and siRNA decreased AFP secretion by PLC/PRF/5 cells. These observations were in agreement with a study conducted by Soresi et al. [66], a clinical study that showed the limited usefulness of AFP in the diagnosis of HCC of viral etiology and that it is more useful for the diagnosis of HCC of non-viral etiology. Based on our *in vitro* results and the previous studies on AFP expression in HCC of viral etiology, we decided not to study the *in vivo* effect of costunolide on AFP secretion by PLC/PRF/5 cells in immunodeficient mice.

Cell migration is directly related to metastasis and is the main cause of morbidity and mortality in patients with cancer. Some studies have reported that telomerase activation enhances cell migration in tumor cancer cells, such as fibroblasts [67] and human pancreatic carcinoma cells [68]. In this study, we showed that telomerase inhibition by costunolide and BIBR decreases the migration ability of cells. Vo Bt et al. (2013) [69] reported that the epithelial mesenchymal transition (EMT), an important indicator of cell progression, is induced by TGF-β through activation of the PI3K/Akt/mTOR signaling pathway in prostate cancer cells. In fact, increased cell migration often correlates with a weakening of intercellular interactions, a loss of cell-cell adhesion and alterations in cell polarity [70]. In tumor cells, stimulation of tyrosine kinase receptors leads to dimerization and auto-phosphorylation, which allows binding to SH2-domain-containing molecules, such as PI3K, and thereby the local generation of PIP3. Studies on isolated migrating cells, such as *Dictyostelium* cells and leukocytes, indicate that PI3K and the phosphoinositide phosphatase PTEN initiate front-rear polarity [71]. This finding correlates with our results, showing that the inhibition of mTOR by rapamycin decreases the migration ability of HepG2/C3A cells. PKC activation of the small G-protein Ras and of the Raf-MEK-ERK downstream pathway is also involved in tumor cell migration [72]. Le Dow et al. (2008) [73] reported that in the absence of Scrib, a protein complex involved in cell migration ability, activated Ras alters mammary epithelial cell-cell junctions, leading to increased epithelial cell migration. In fact, Ras activation prevents normal epithelial junction formation and plays a role in the alteration of polarity protein localization. In our study, we confirmed that the inhibition of PKC decreases cell migration ability in HCC. However, we did not examine whether this effect is due to Ras/Raf/MEK/ERK pathway signaling inhibition or to another pathway or mechanism. Cell invasion is another hallmark of cancer and is the main cause of mortality in cancer patients. In human glioblastoma cell lines, cell invasion is inhibited by the knockdown of hTERT, showing the direct relationship between telomerase activity and cell invasion in cancer [74]. Furthermore, the overexpression of STAT3 may activate cell invasion through the regulation of MMPs, VEGF or E-cadherin and may occur through JAK/STAT3 activation or by interaction with other transduction pathways, such as the PI3K/AKT pathway and the activation of other STAT family members [75,76]. Cell invasion is also regulated by PKC. In fact, activated PKC isoforms induce the secretion of MMP-9, leading to the activation of MMP-2, downregulate TIMP-1 and TIMP-2 secretion, and increase MT1-MMP on the cell surface [77]. These findings correlate with our results because telomerase, STAT3 and PKC inhibition decreased the invasive potential of PLC/PRF/5 cells.

Despite its role as a hepatocellular carcinoma serum marker, the role of AFP in cancer growth and metabolism has been investigated, and great progresses have been made. It has been demonstrated that AFP promotes tumor progression [78], affects cell differentiation, growth regulation, tumorigenesis and is involved in pleiotropic activities [78,79]. In addition, the overexpression of AFP promotes the proliferation of normal and cancer cells, which indicates that AFP may be involved in cancer liver progression [80]. However, the treatment of cells with telomerase or signaling pathway inhibitors did not show any negative effect on cell proliferation at least on a short treatment time. This is likely due to the relative short treatment time. This role of AFP in cell proliferation appears to be driven through one of the most activated signaling pathways in cancer, the PI3K/AKT pathway [81]. The AFP telomerase also plays an extratelomeric role in cancer cells. However, it has been found that telomerase upregulates several genes that promote cell growth and downregulates seven growth-inhibitory genes [74]. In addition, hTERT was previously shown to be involved in the modulation of glycolytic enzymes and resistance to apoptosis and is linked to cancer metastasis [82]. Thus, we aimed to investigate the pathway used by telomerase to modulate AFP expression and secretion. The use of three different inhibitors for PI3k and other inhibitors for mTOR and STAT3 clearly showed that the PI3k/AKT/mTOR/STAT3 signaling pathway is involved in AFP modulation. STAT3 is known to be an activator of transcriptional factors that can directly modulate the AFP gene and its transcription and then the translation and secretion of this protein. A recent study reported that AFP activates the PI3K/Akt/mTOR pathway in HCC cells [83]. In this study, we also demonstrated this interrelationship by showing that the inhibition of the PI3K/Akt/mTOR signaling pathway decreases AFP production. The inhibition of the MAPK/ERK pathway did not significantly alter AFP secretion. Surprisingly, the PKC inhibitor, which usually modulates the MAPK/ERK pathway, had a significant effect on AFP secretion and appeared to not involve the MAPK/ERK pathway. Based on these results, we can conclude that the PI3k/AKT/mTOR/STAT3 pathway directly modulates AFP gene and protein and that PKC directly phosphorylates the hTERT [84] subunit of the telomerase and modulates the AFP gene because the activation of PKC by PMA increases the secretion of AFP. The combination of the two telomerase inhibitors used in this study with the inhibitors of the signaling pathway PI3k/AKT/mTOR/STAT3 gave, unexpectedly, different results. The BIBR 1532, costunolide and the inhibitors of PI3K/AKT/mTOR decreased AFP expression and secretion; however, only costunolide had an additive effect when combined with the signaling pathway inhibitors. Although, the costunolide is a major telomerase inhibitor, it is also known to the drop the intracellular glutathione (GSH) and consequently inhibits tyrosine-phosphorylation of STAT3 in HepG2/C3A [85]; In addition, costunolide inhibits c-Fos and NF-kb, both related to PI3k/Akt/mTOR signaling pathway modulation; This could explain the additional effect of this molecule shown in this study. Our results did not definitely determine whether telomerase inhibits AFP expression and secretion through the PI3k/AKT/mTOR signaling pathway or through another independent pathway. However, because the STAT3 inhibitor showed the strongest effect on AFP secretion, we hypothesize that telomerase acts through different signaling pathways, which converge with the PI3k/AKT/mTOR signaling pathway to activate STAT3.

**Fig 12:**
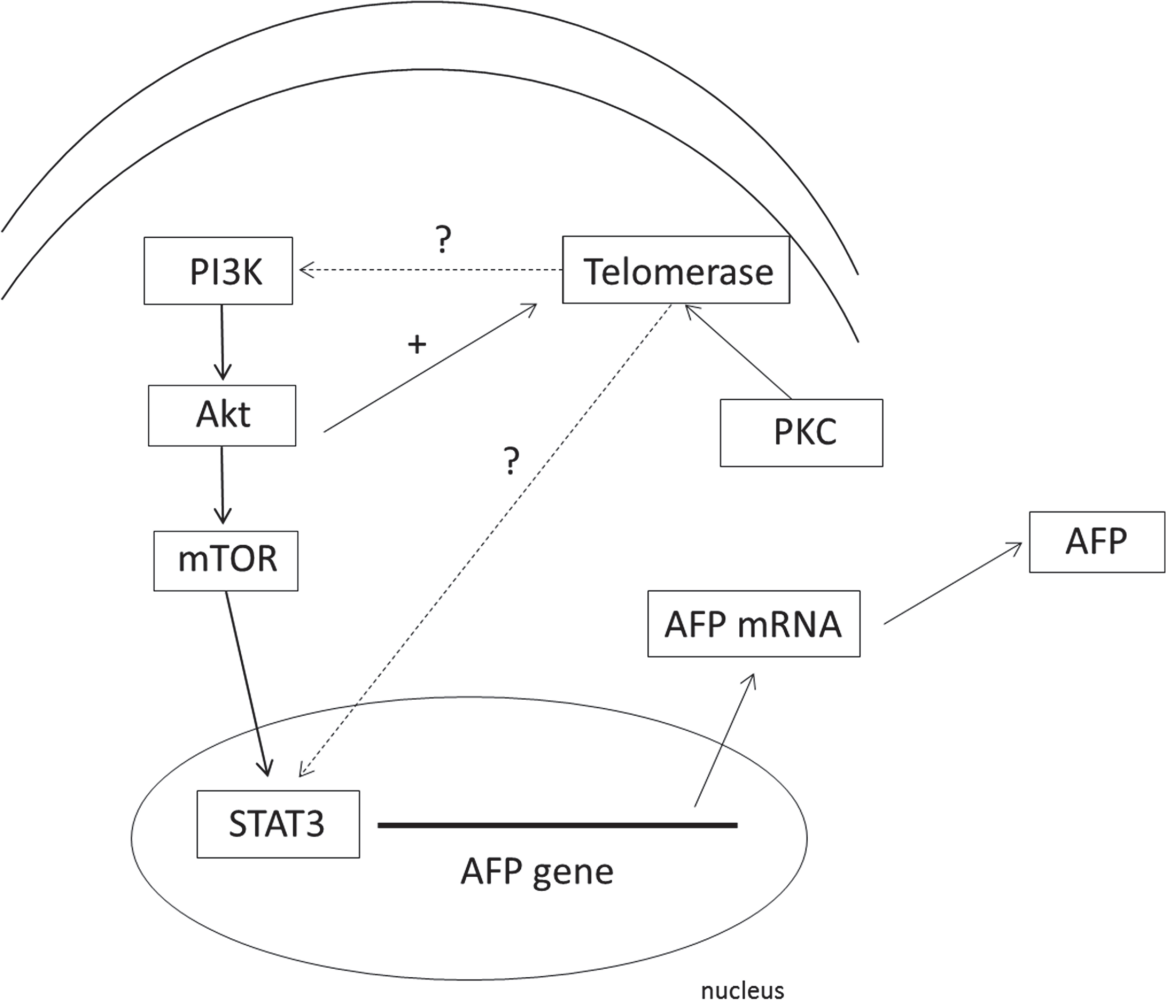
Schematic representation of intracellular signaling pathways leading to AFP expression and secretion. Our study demonstrates that the inhibition of telomerase, PKC and the PI3k/Akt/mTOR/STAT3 signaling pathway leads to decreases in AFP expression and secretion. In contrast, the inhibition of the MAPK/ERK pathway did not show any effect. However, the combination of a telomerase inhibitor with other pathway inhibitors (see results section) presented an additive effect with costunolide. The mechanism underlying the action of telomerase remains unclear. The modulation of AFP by telomerase may be either through the PI3k/Akt/mTOR/STAT3 signaling pathway or through an independent pathway. Further investigations are needed to elucidate the modulation of AFP by telomerase.

In conclusion, our study provides the first demonstration that telomerase inhibition decreases AFP expression and secretion in HCC. The action of these two inhibitors may involve the activation of either the cell survival PI3k/AKT/mTOR signaling pathway or another pathway that activate the gene regulator STAT3 “Fig. 12”. The inclusion of telomerase inhibitors in a chemotherapy protocol for patients with hepatocellular carcinoma may have a negative effect on cell proliferation and immortalization by inhibiting telomere repair, by inhibiting the extra-telomeric cell surviving effect of telomerase, and by targeting AFP production, which is a growth factor by itself; however, more *in vivo* animal and clinical studies should be performed. In future studies, we will assess the effect of long-term treatment and monitoring using a telomerase inhibitor on mice well-being and survival, even though there was no effect on tumor volume. This study will allow us to evaluate the individual effect of decreasing AFP without considering the tumor volume. We will also investigate the role of C/EBP, a common transcription factor for AFP and hTERT genes [23,55] and explore the role of PKC on telomerase protein accessories, such as AP1, on AFP modulation.
